# Supplementary material for: Intracellular aggregation of the transthyretin protein is limited by Hsp70 chaperones
Source: bioRxiv. 2025 Dec 16:2025.12.14.694244. Preprint. [Version 1] doi: 10.64898/2025.12.14.694244 (PMC12724544; doi:10.64898/2025.12.14.694244)
Supplement: Supplement 1 [file media-1.pdf]

Title: Intracellular aggregation of the transthyretin protein is limited by Hsp70 chaperones

Claire M. Radtke[1], Adam S. Knier[1,2], Sean A. Martin[1], Jane E. Dorweiler[1], Matt E. Hudson[,3,4] and Anita L. Manogaran[1]*

1 Department of Biological Sciences, Marquette University, Milwaukee, WI, 53201-1881 USA

2 Department of Biochemistry, Medical College of Wisconsin, Milwaukee, WI 53226, USA

3 Department of Crop Sciences, University of Illinois at Urbana-Champaign, 1102S Goodwin Ave, Urbana, IL, 61801, USA

4 Carl R. Woese Institute for Genomic Biology, University of Illinois at Urbana-Champaign, Urbana, IL 61801, USA

* Corresponding Author

Running title: Hsp70 limits TTR aggregation

Keywords: protein aggregation, molecular chaperones, Hsp70, disaggregation, proteostasis, transthyretin

Supporting Experimental Procedures

Serial Dilution Plating Assay

Transformants were patched on solid selective media and grown overnight at 30°C. Fresh patches were used to inoculate 4 ml cultures in plasmid selective media. Strains were grown at 30˚C, normalized for cell density, spotted onto selective media in 5-fold serial dilutions, and incubated at 30˚C for several days.

Generation of Sse1^{WT} and Sse1^{G233D} plasmid

The *SSE1* gene, including its endogenous promoter, coding sequence, and terminator, was PCR-amplified from genomic DNA from the 74D-694 background using gene-specific primers listed in Supplementary Table 3. The purified PCR product was introduced into the pGEM-T Easy vector (Promega) and verified by *EcoRI* and *BglII* digestion. Site-directed mutagenesis using the Q5® Site-Directed Mutagenesis Kit (New England Biolabs, E0554) was performed to generate the Sse1^{G233D} point mutation. Clones were sequenced for verification. Wildtype and mutant *SSE1* constructs were introduced into the pRS314 yeast expression plasmid by *ClaI* digestion. Successful clones were confirmed by colony PCR and/or restriction digest analysis.

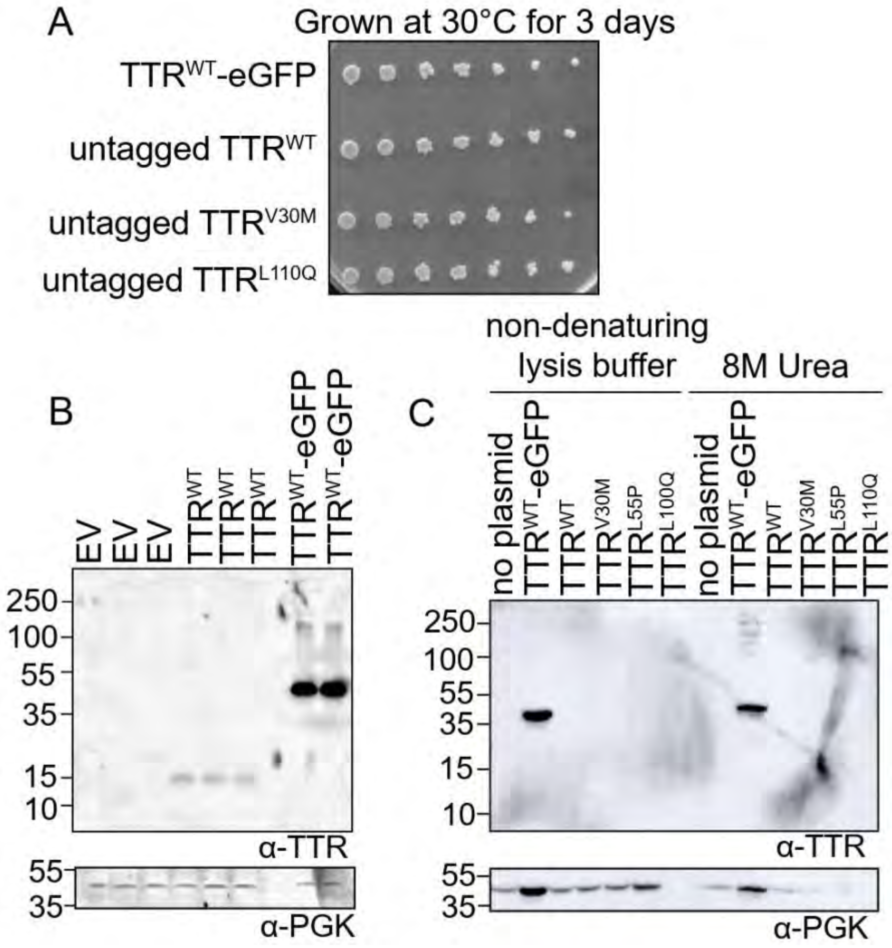

Supplemental Figure 1. Untagged TTR exhibits low to no detection compared to TTR-eGFP. A) 74-D694 wildtype strains (D230) were transformed with TTR^{WT}-eGFP (p3146), untagged TTR^{WT} (p3235), untagged TTR^{V30M} (p3240), and untagged TTR^{L110Q} (p3244). Cells were serially diluted 5-fold, spotted on SD-Ura plates, and grown at 30°C for three days. Shown is a representative image from two independent trials. B) Wildtype strains were transformed with empty vector (EV, p3299), untagged TTR^{WT} (p3301), and TTR^{WT}-eGFP. Samples were analyzed by SDS-PAGE and immunoblotted with polyclonal anti-TTR or anti-PGK antibodies. C) Wildtype strains were transformed with TTR-eGFP, untagged TTR^{WT}, untagged TTR^{V30M}, untagged TTR^{L55P} (p3242), and untagged TTR^{L110Q}. Cultures were split in two and lysates isolated either in standard non-denaturing 1X lysis buffer or in the presence of 50mM HEPES pH 7.4 and 8M urea. Samples were analyzed by SDS-PAGE and immunoblotted with the indicated monoclonal anti-TTR or anti-PGK antibodies.

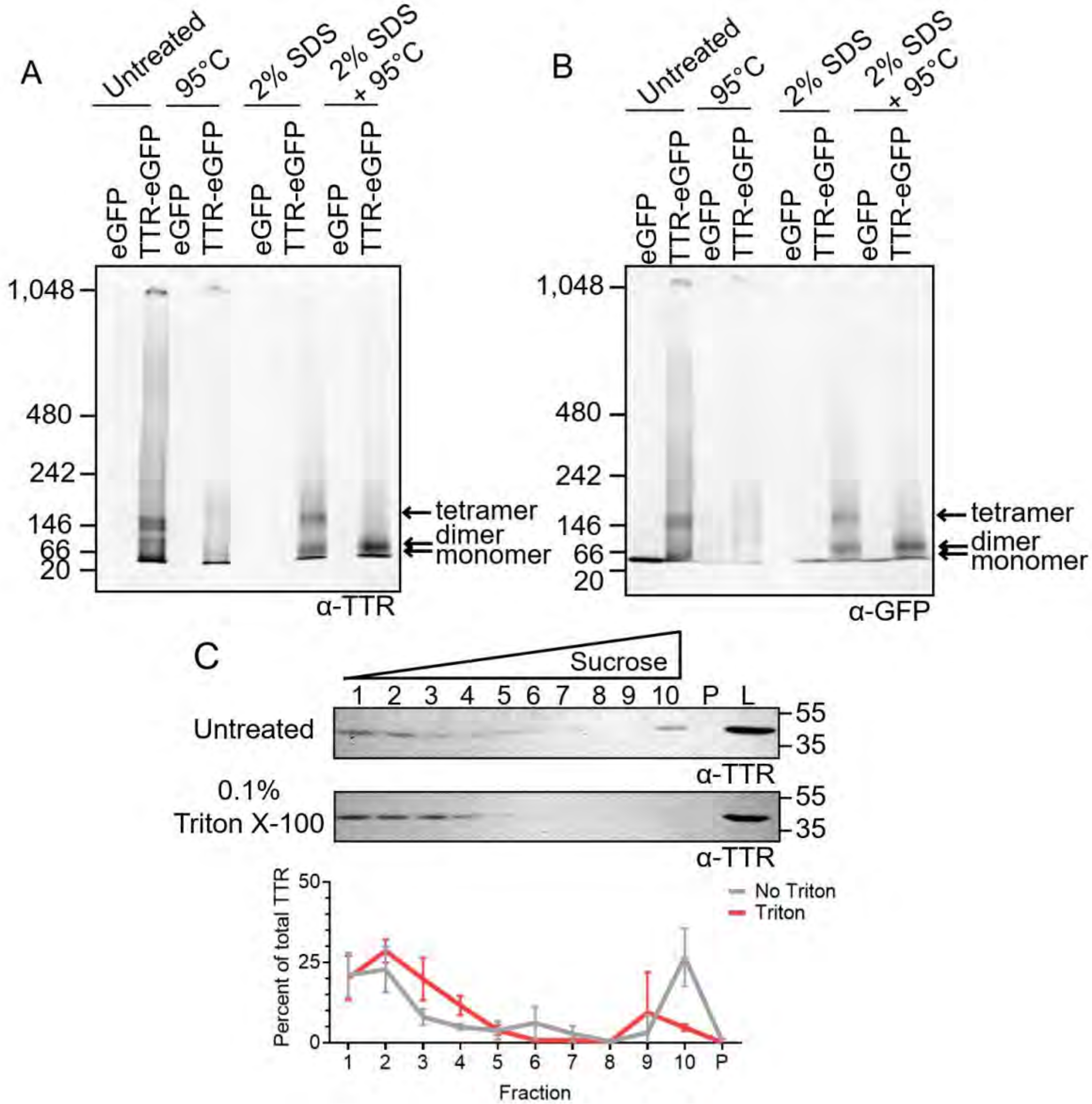

Supplemental Figure 2. Tris-acetate native PAGE analysis and triton X-100 solubility of TTR-eGFP. Wildtype strains were transformed eGFP or TTR-eGFP. Lysates were untreated, incubated at 95°C for 8 minutes, treated with 2% SDS, or 2% SDS and incubated at 95°C for 8 minutes. Lysates were analyzed by 3-8% tris-acetate native PAGE and immunoblotted with A) polyclonal anti-TTR or B) anti-GFP. C) Lysates from wildtype strains transformed TTR-eGFP were split and either left untreated or treated with 0.1% Triton X-100 for 10 minutes prior to loading on the sucrose gradient. All fractions were analyzed by SDS-PAGE and immunoblotted with a monoclonal anti-TTR antibody. Shown is a representative of three independent biological trials. The TTR signal from each fraction was normalized to the combined TTR signal in fractions 1-10 and pellet and graphed as the mean ± SD. Statistical analyses were performed with a two-way repeated measures ANOVA, Geisser-Greenhouse correction, and a Sidak multiple comparisons test (* $p < 0.05$). No comparisons are significant.

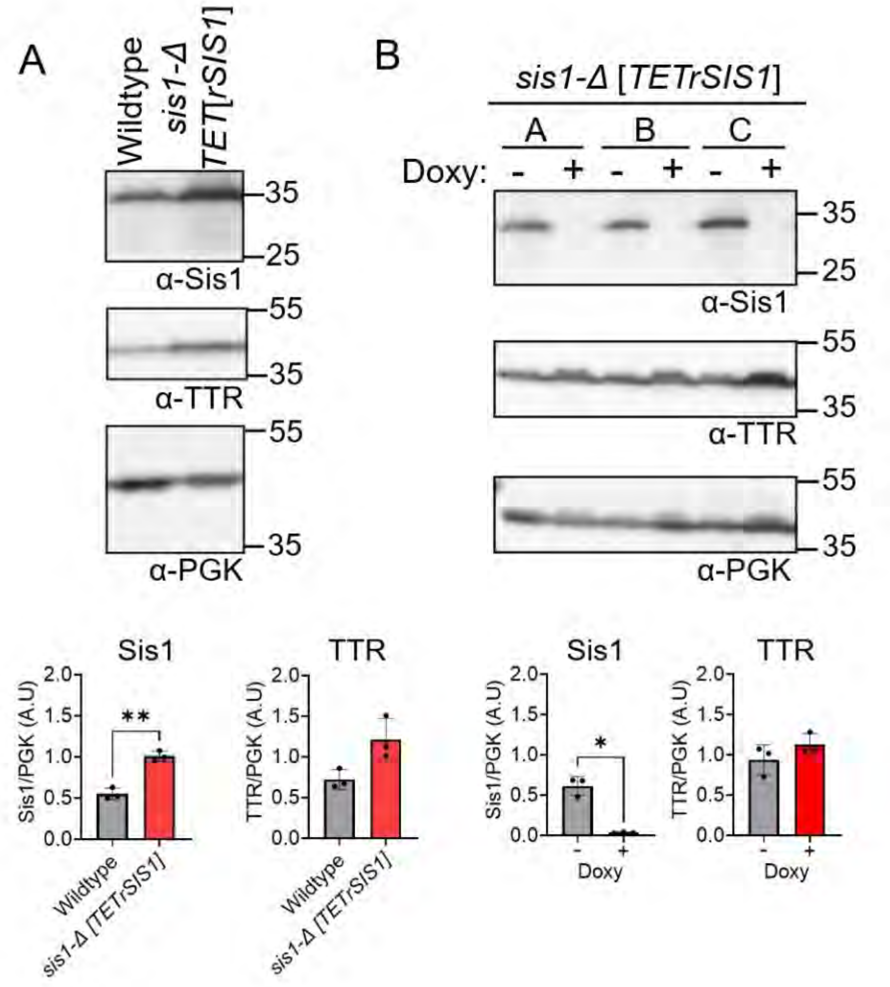

Supplemental Figure 3. Doxycycline reduces tetracycline-repressible Sis1 levels by approximately 17-fold. A) Wildtype and *sis1-Δ* [*TETrSIS1*] strains were transformed with TTR-eGFP. Untreated lysates were analyzed by SDS-PAGE and immunoblotted with the indicated antibodies. Statistical analyses were performed with an unpaired t-test (** $p < 0.01$). B) *sis1-Δ* [*TETrSIS1*] strains were transformed with TTR-eGFP. Cultures were split into untreated and treated with 10 μg/mL doxycycline overnight. Lysates from three independent replicates (A-C) were analyzed by SDS-PAGE followed by immunoblotted with the indicated antibodies. Statistical analyses were performed using paired t-test (* $p < 0.05$). All graphs have dots as independent trials and graphed as means ± SD. Unlabeled comparisons are not significant.

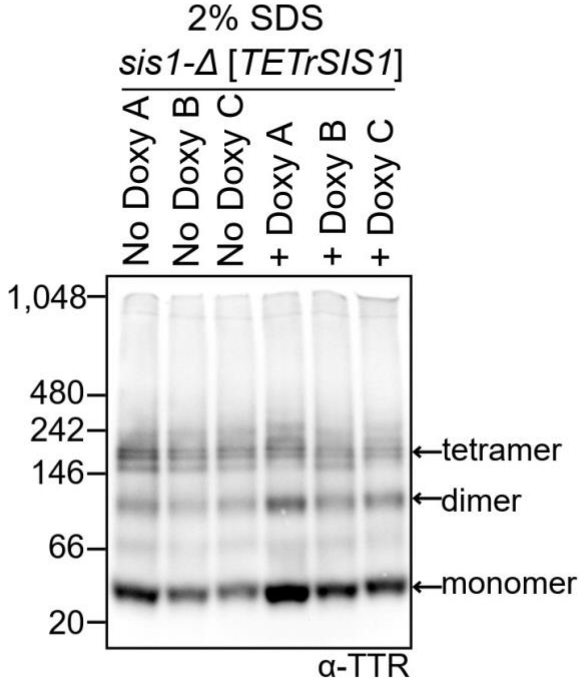

Supplemental Figure 4. SDS treated *sis1-Δ* [*TETrSIS1*] shows no difference in TTR-eGFP on native PAGE. *sis1-Δ* [*TETrSIS1*] cultures expressing TTR-eGFP were split and either left untreated or treated with 10 µg/mL doxycycline overnight. Lysates were treated with 2% SDS prior to 4–20% tris-glycine native PAGE and immunoblotted in polyclonal anti-TTR antibody. Estimated monomeric, dimeric, and tetrameric TTR-eGFP sizes are labeled.

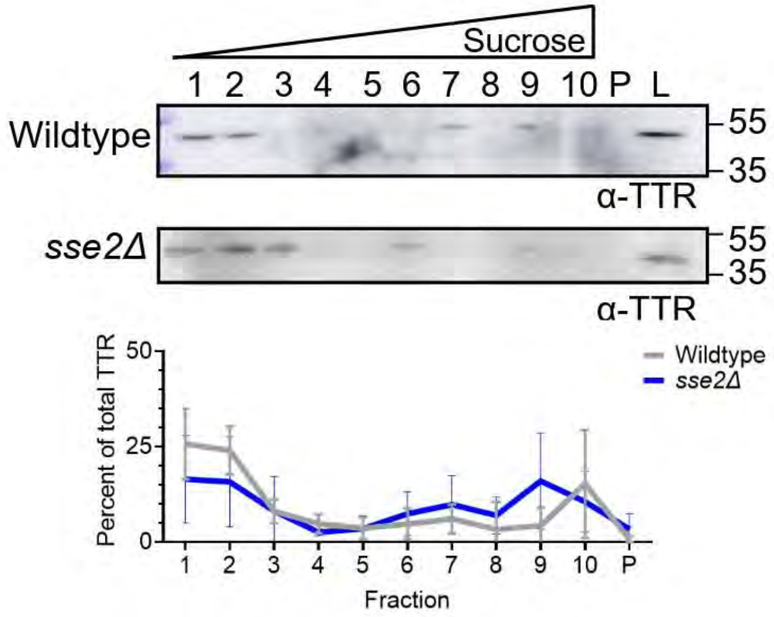

Supplemental Figure 5. Deletion of Sse2 does not alter TTR-eGFP sedimentation. Lysates from *sse2Δ* (M650) cultures expressing TTR-eGFP were loaded onto discontinuous sucrose gradients (10%, 40%, and 60%). Individual fractions, pelleted protein (P), and whole-cell lysates (L) were analyzed by SDS-PAGE and immunoblotted with a monoclonal anti-TTR antibody. Shown is a representative blot from four independent trials. The TTR signal from each fraction was normalized to the combined TTR signal in fractions 1-10 and pellet and graphed as mean ± SD. Statistical analyses were performed with a two-way repeated measures ANOVA, Geisser-Greenhouse correction, and a Sidak multiple comparisons test (* $p < 0.05$). No comparisons are significant.

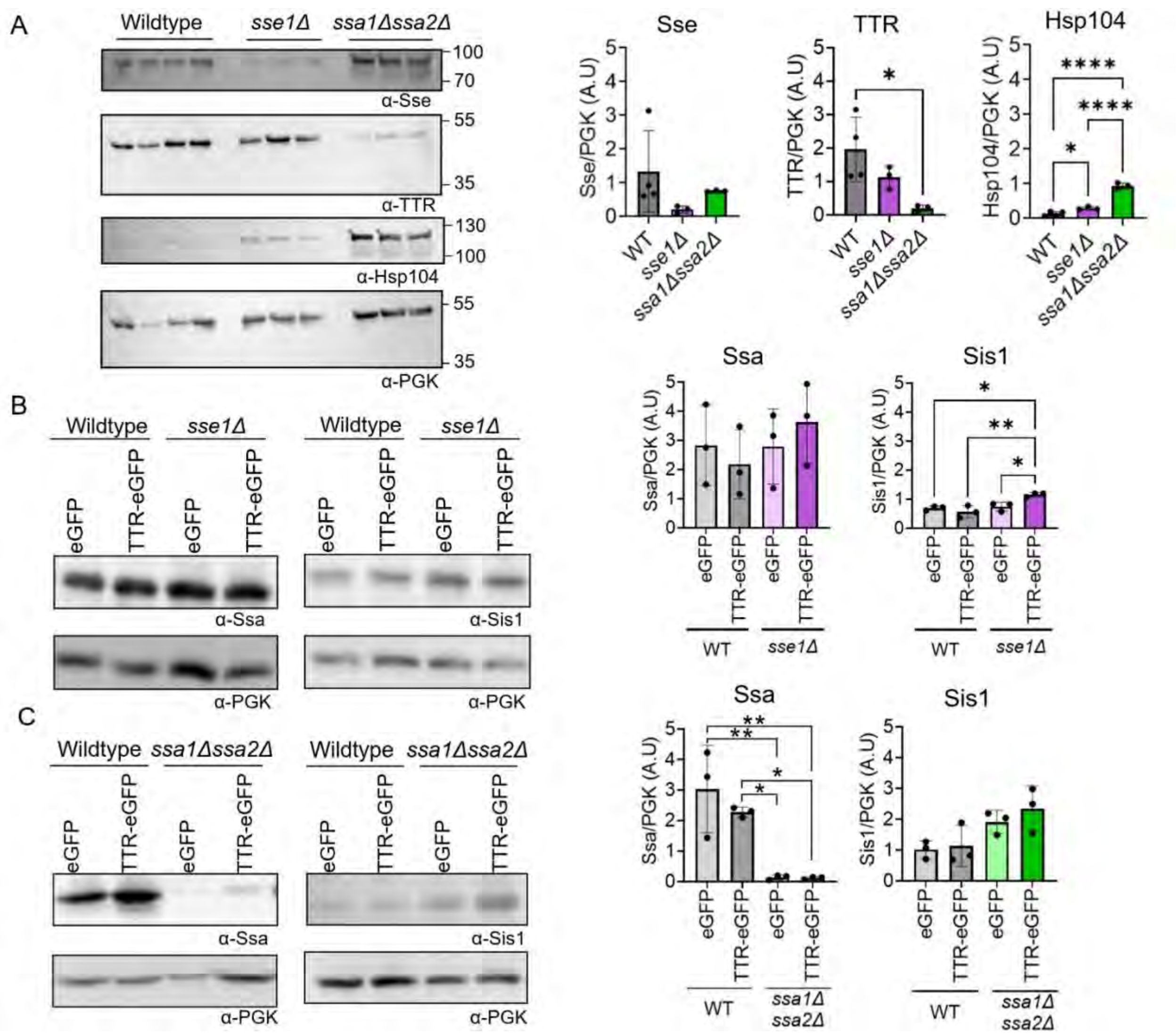

Supplemental Figure 6. Chaperone and TTR steady state levels in *sse1Δ and ssa1Δssa2Δ* strains*.* A) Lysates from wildtype (gray bars on graphs), *sse1Δ* (purple bars)*, and ssa1Δssa2Δ* (green bars) strains transformed with TTR-eGFP were analyzed via SDS-PAGE and immunoblotted with monoclonal anti-TTR, anti-Hsp104, anti-Sse (recognizes Sse1 and Sse2) and anti-PGK antibody. B) Wildtype and *sse1Δ* strains subjected to immunoblotting against anti-Ssa (recognizes Ssa1-4), and anti-Sis1 antibodies. C) Same as B except for *ssa1Δssa2Δ* strains. All graphs quantify the steady state levels normalized to PGK. Statistical analyses were performed with a one-way ANOVA and Tukey post hoc test (* $p < 0.05$, ** $p < 0.01$, **** $p < 0.0001$). Unlabeled comparisons are not significant. Dots indicate independent trials, graphed as mean ± SD.

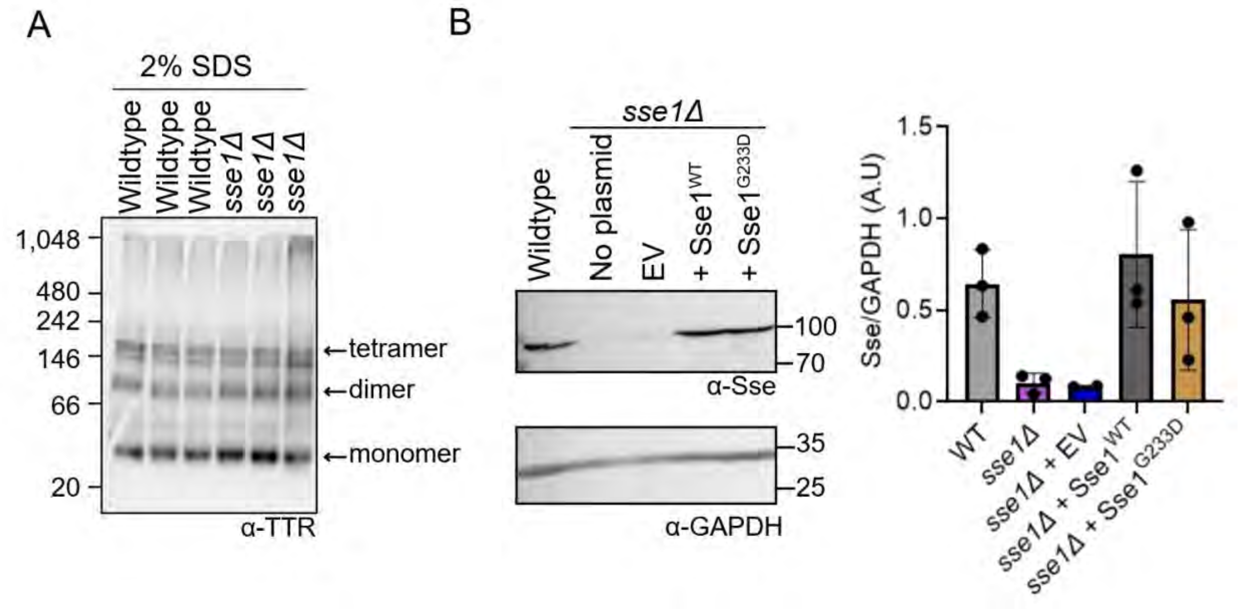

Supplemental Figure 7. SDS treated *sse1Δ* native PAGE and Sse1 variant steady state levels. A) Lysates *sse1Δ* strain expressing TTR-eGFP were treated with 2% SDS prior to loading onto 4–20% tris-glycine native PAGE and immunoblotted in polyclonal anti-TTR antibody. Estimated monomeric, dimeric, and tetrameric TTR-eGFP sizes are labeled. B) Lysates of wildtype strains or *sse1Δ* strains expressing either wildtype Sse1 (p3360), Sse1^{G233D} mutant (p3362), or empty vector (p3114) were transformed with TTR-eGFP. Lysates were subjected to Western blot analysis to determine Sse1 steady state levels normalized to GAPDH. Dots indicate independent trials graphed as mean ± SD. Statistical analysis was performed with a one-way ANOVA and Tukey post hoc test (* $p < 0.05$). No comparisons are significant. Dots indicate independent trials graphed as mean ± SD.

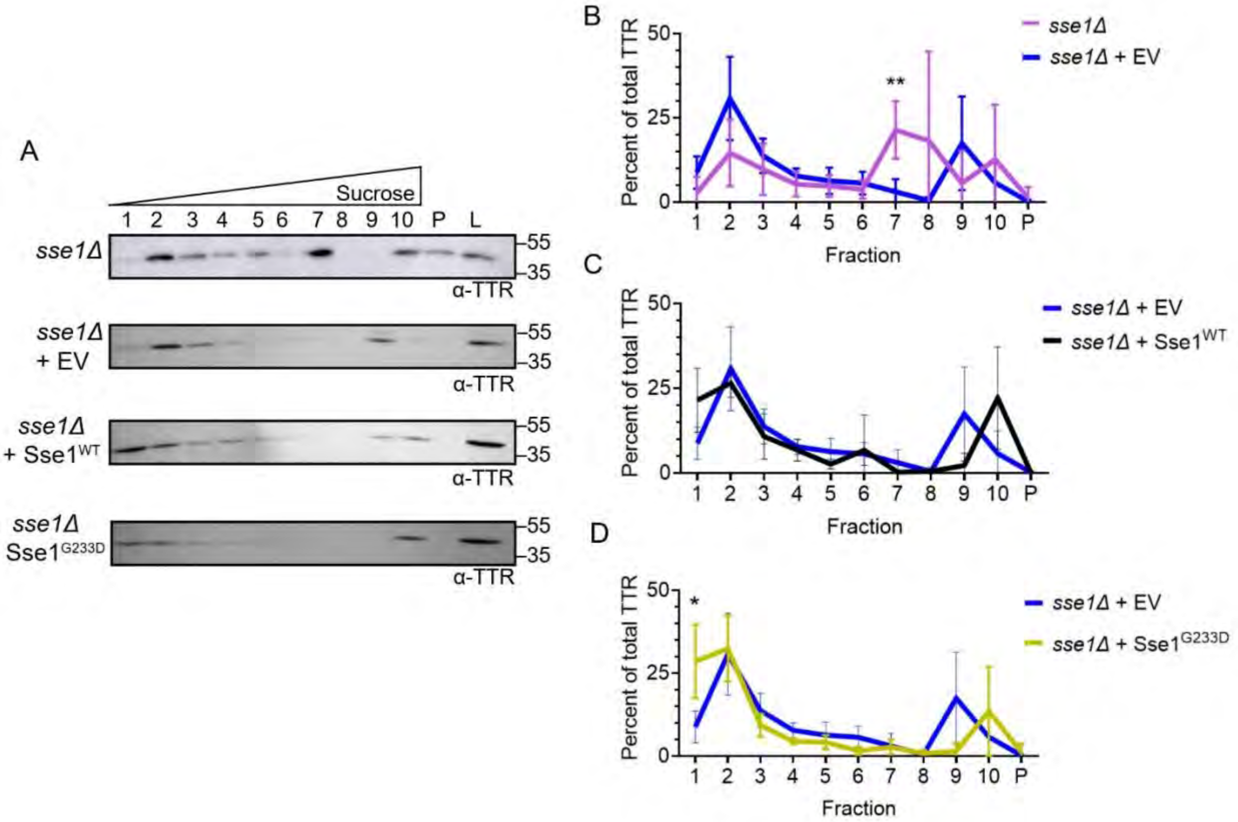

Supplemental Figure 8. TTR-eGFP is detected in fraction 1 in both *sse1Δ* strains expressing Sse1^{WT} and Sse1^{G233D}. *sse1Δ* strains (M649) were transformed with plasmids expressing either empty vector (p3114), wildtype Sse1 (p3360), or mutant Sse1^{G233D} (p3362) and TTR-eGFP. Sucrose gradient fractionation was performed, and fractions were subjected to Western blot analysis using monoclonal anti-TTR antibody. A) Representative sucrose gradient images of the indicated strains are shown. B) Quantification of the TTR signal from each fraction normalized to the combined TTR signal in fractions 1-10 and pellet of *sse1Δ* (n=6) vs. *sse1Δ* + empty vector (EV; n=5). The mean ± SD is graphed. Statistical analyses were performed with a two-way repeated measures ANOVA, Geisser-Greenhouse correction, and a Sidak multiple comparisons test (* $p < 0.05$). C) Same as B, except *sse1Δ* + EV to *sse1Δ* + Sse1^{WT} (n=4), and D) *sse1Δ* + EV to *sse1Δ* + Sse1^{G233D} (n=4). Unlabeled comparisons are not significant.

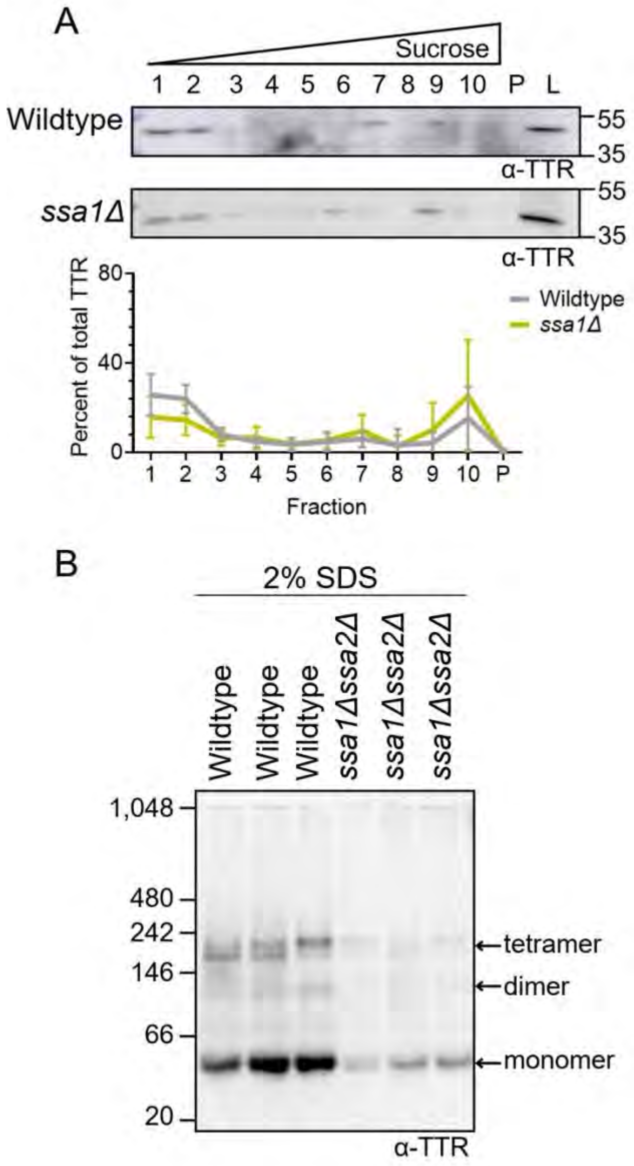

Supplemental Figure 9. *ssa1Δ* shows no change in TTR-eGFP sedimentation and SDS treatment shows no change in TTR-eGFP in *ssa1Δssa2Δ* native PAGE. A) Lysates from *ssa1Δ* strains (M652) expressing TTR-eGFP were subjected to discontinuous sucrose gradient centrifugation (10%, 40%, and 60%). Fractions were analyzed by SDS-PAGE and immunoblotted with a monoclonal anti-TTR antibody. Shown is a representative image of five independent trials. The percent of TTR signal normalized to the combined TTR signal in all fractions was quantified and graphed as mean ± SD. Statistical analyses were performed with a two-way repeated measures ANOVA, Geisser-Greenhouse correction, and a Sidak multiple comparisons test (* $p < 0.05$). No comparisons are significant. B) Lysates from *ssa1Δssa2Δ* strains expressing TTR-eGFP were treated with 2% SDS prior to 4–20% tris-glycine native PAGE and immunoblotted with polyclonal anti-TTR antibody. Estimated monomeric, dimeric, and tetrameric TTR-eGFP sizes are labeled.

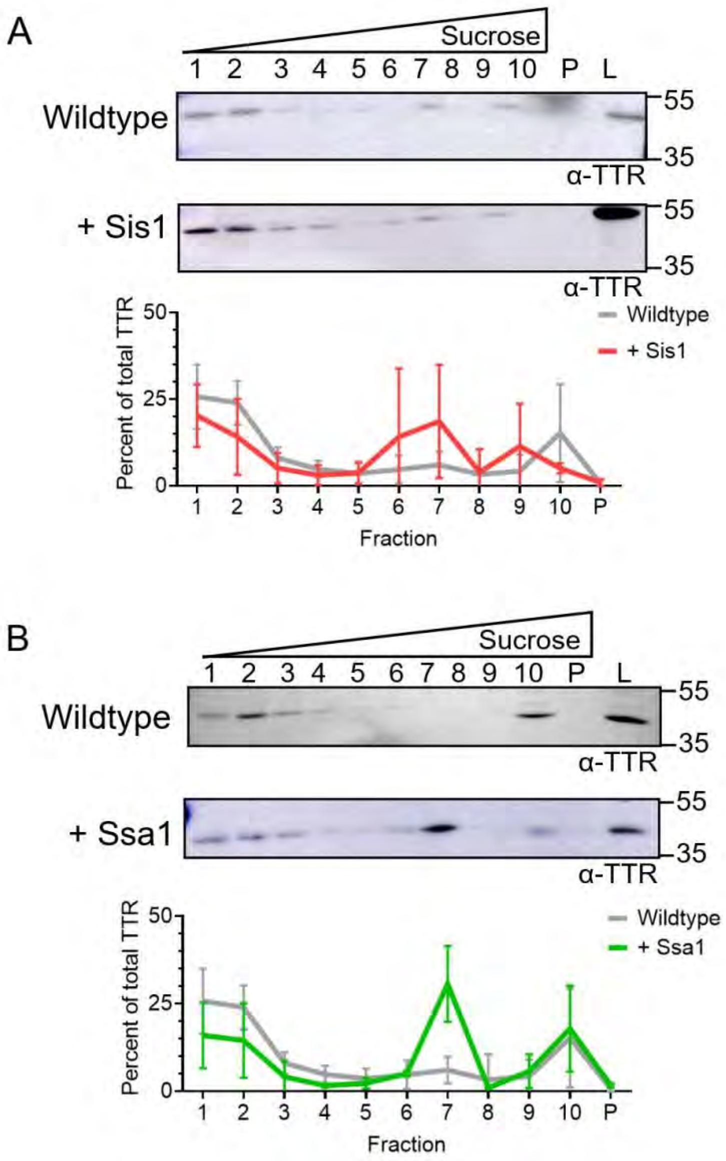

Supplemental Figure 10. No significant changes detected in TTR-eGFP sedimentation upon Sis1 and Ssa1 overexpression. Wildtype strains transformed with TTR-eGFP and A) GPD-Sis1 (p3347) or B) GPD-Ssa1 (p3302) were subjected to discontinuous sucrose gradient sedimentation. Fractions were subjected to Western blot analysis using a monoclonal anti-TTR antibody. Shown are representative blots from three independent trials each. The TTR signal from each fraction was normalized to the combined TTR signal in fractions 1-10 and pellet, and the mean ± SD is graphed. Statistical analyses were performed with a two-way repeated measures ANOVA, Geisser-Greenhouse correction, and a Sidak multiple comparisons test (* $p < 0.05$). No comparisons are significant.

Supplementary Table 1. Yeast strains used in this study

Genetic background	Strain number	Genotype	Name in manuscript	Reference
74-D694	D230	*MAT**a** ade1-14 ura3-52 leu2-3,112 trp1-289 his3-200 [psi$^-$][pin$^-$]*	Wildtype	(Chernoff, Lindquist et al. 1995)
74-D694	M649	*MAT**a** ade1-14 ura3-52 leu2-3,112 trp1-289 his3-200 sse1::HIS3 [psi-] [pin-]*	*sse1Δ*	This study
74-D694	M650	*MAT**a** ade1-14 ura3-52 leu2-3,112 trp1-289 his3-200 sse2::HIS3 [psi-] [pin-]*	*sse2Δ*	This study
74-D694	M652	*MAT**a** ade1-14 ura3-52 leu2-3,112 trp1-289 his3-200 ssa1::HIS3 [psi-] [pin-]*	*ssa1Δ*	This study
74-D694	M674	*MAT**a** ade1-14 ura3-52 leu2-3,112 trp1-289 his3-200 ssa1::HIS3 ssa2::NatR [psi-] [pin-]*	*ssa1Δ ssa2Δ*	(Buchholz, Martin et al. 2025)
74-D694	M677	*MAT**a** ade1-14, ura3-52, leu2-3,112, trp1-289, his3-200, Δsis1::LEU2, TetR-Sis1 74D-694 (TRP plasmid) [pin-]*	*sis1-Δ* [*TETrSIS1*]	(Hines, Higurashi et al. 2011)

Supplemental Table 2. Plasmids Used in this Study:

Plasmid number	Plasmid name	Yeast Marker	Name in manuscript	Reference
p3141	pAG426GPD-ccdB-EGFP	URA3 (2 micron)	EV-GFP	Addgene plasmid #14204 deposited by Susan Lindquist
p3146	pAG426GPD-TTR-WT-EGFP	URA3 (2 micron)	TTR-GFP	(Knier, Davis et al. 2022)
p3299	pAG425GPD-ccdB	LEU2 (2 micron)	Empty vector for untagged TTR	Addgene plasmid #14154; deposited by Susan Lindquist
p3301	pAG425GPD-TTR	LEU2 (2 micron)	Untagged TTR^{WT}	This study
p3235	pAG426GPD-TTR-WT	URA3 (2 micron)	Untagged TTR^{WT}	This study
p3240	pAG426GPD-TTR-V30M	URA3 (2 micron)	Untagged TTR^{V30M}	This study
p3242	pAG426GPD-TTR-L55P	URA3 (2 micron)	Untagged TTR^{L55P}	This study
p3244	pAG426GPD-TTR-L110Q	URA3 (2 micron)	Untagged TTR^{L110Q}	This study
p3302	pAG415-GPD-Ssa1	Leu2, (CEN)	GPD-Ssa1	This study
p3114	pRS314	TRP1 (CEN)	Empty vector for pRS314 $Sse1^{WT}$ and $Sse1^{G233D}$	(Sikorski and Hieter 1989)
p3360	pRS314-$Sse1^{WT}$	TRP1 (CEN)	\$Sse1^{WT}$	This study
p3362	pRS314-$Sse1^{G233D}$	TRP1 (CEN)	$Sse1^{G223D}$	This study

Supplementary Table 3 – Antibodies used in this study

Antibody	Dilution	Clonality	Vendor	Identifier
Pre-albumin	1:1000	Monoclonal	Santa Cruz Biotechnology	Cat # sc-377517
TTR 1-147	1:1000	Polyclonal	Invitrogen	Cat # PA5-27220
Hsp104	1:2000	Polyclonal	Enzo Life Sciences	Prod. No. ADI-SPA-1040
Phosphoglycerate Kinase	1:1000	Monoclonal	Novex by Life Technologies	Cat # 459250
GAPDH	1:1000	Monoclonal	Invitrogen	Cat # MA5-15738
Sis1	1:10,000	Polyclonal	Craig Lab	#66932
Ssa1-4	1:10,000	Polyclonal	Craig Lab	#1173
Green Fluorescent Protein	1:5000	Monoclonal	Sigma	Cat # G1546
Anti-Mouse, AP	1:10,000	N/A	Sigma Life Sciences	SKU A3562-.5ML
Anti-Mouse, HRP	1:10,000	N/A	Sigma Life Sciences	SKU A9044-2ML
Anti-Rabbit, HRP	1:10,000	N/A	Sigma Life Sciences	SKU A9169-2ML

Supplementary Table 4. Primers used in this study

Generated	ID	Name	Sequence (5'-3')
sse1Δ	AM532	AM532_Sse1Δ sense primer	CCATAAGCAAAAAGTACATTGACA AACAACATTTCTTTAAAAGATGACA GAGCAGAAAGCCCTAGTAAAGC
sse1Δ	AM533	AM533_Sse1Δ antisense primer	CGGAAAAAACAATAAAGATCCTTTT CTAGTTACTTTGCTGCATTAACACT ACATAAGAACACCTTTGGTGG
sse1Δ	AM547	AM547_Sse1diagnostic 4deletion	CCATTTTAAACTCCCTCTGTC
sse2Δ	AM558	AM558_Sse2_sense_deletion	TTTTTTACCTGTAACAGACGTAACC AAAGGATATAATATAATGACAGAGC AGAAAGCCCTAGTAAAGC
sse2Δ	AM559	AM559_Sse2_antisense_deletion	AGAATAAAGAGGGAACAATCCAAA TAGACAAAAATTCCGACTACATAAG AACACCTTTGGTGG
sse2Δ	AM561	AM561_Sse2_sense_diagnostic4deletion	GCCGTTTAGAGATTTTTATTTATG
ssa1Δ	AM553	AM553_Ssa1_sense_deletion	GTATTACAAGAAACAAAAATTCAAG TAAATAACAGATAATATGACAGAGC AGAAAGCCCTAGTAAAGC
ssa1Δ	AM554	AM554_Ssa1_antisense_deletion	GACATTTTCGTTATTATCAATTGCC GCACCAATTGGCCTACATAAGAAC ACCTTTGGTGG
ssa1Δ	AM560	AM560_Ssa1_sense_diagnostic4deletion	CGTTTCCCAATTCTTACTTAAG
ssa1Δssa2Δ	AM584	AM584_Ssa2_NatRdisruption_sense	CCAACAGATCAAGCAGATTTTATAC AGAAATATTTATACAATGGGTACCA CTCTTGACG
ssa1Δssa2Δ	AM585	AM585_Ssa2_NatRdisruption_antisense	AGTAAAACTTTTCGGATATTTTACA GGGCGATCGCTAAGCTTAGGGGC AGGGCATGCTC
ssa1Δssa2Δ	AM220	NATr AS1	AAGACGGTGTCGGTGGTGAAGG
ssa1Δssa2Δ	AM422	AM422_Ssa2_fwd_at -269	CCGAGAAGTTCTTCCGATTCAC
$Sse1^{WT}$	AM432	AM432_Sse1_fwd_at -747	ACGGTAGGAGATTTGCCACTG
$Sse1^{WT}$	AM433	AM433_Sse1_rev_200_bp_3'_of_stop	GGGTTTAGGAGACTAATGCGTG
$Sse1^{G233D}$	AM625	AM625_Sse1_G233D_Mut_Sense	CAAGCATTTTGATGGTAGAGACTT C
$Sse1^{G233D}$	AM626	AM626_Sse1_G233D_Mut_Anti	TCGCAGGCAGTTCCT

References:

1. Chernoff, Y. O., S. L. Lindquist, B. Ono, S. G. Inge-Vechtomov and S. W. Liebman (1995). "Role of the chaperone protein Hsp104 in propagation of the yeast prion-like factor [psi+]." Science **268**(5212): 880–884.
2. Buchholz, H. E., S. A. Martin, J. E. Dorweiler, C. M. Radtke, A. S. Knier, N. B. Beans and A. L. Manogaran (2025). "Hsp70 chaperones, Ssa1 and Ssa2, limit poly(A) binding protein aggregation." Mol Biol Cell **36**(6): ar66.
3. Hines, J. K., T. Higurashi, M. Srinivasan and E. A. Craig (2011). "Influence of prion variant and yeast strain variation on prion-molecular chaperone requirements." Prion **5**(4): 238-244.
4. Sikorski, R. S. and P. Hieter (1989). "A system of shuttle vectors and yeast host strains designed for efficient manipulation of DNA in Saccharomyces cerevisiae." Genetics **122**(1): 19-27.
